# Study on the Reparative Effect of PEGylated Growth Hormone on Ovarian Parameters and Mitochondrial Function of Oocytes From Rats With Premature Ovarian Insufficiency

**DOI:** 10.3389/fcell.2021.649005

**Published:** 2021-03-15

**Authors:** Penghui Feng, Qiu Xie, Zhe Liu, Zaixin Guo, Ruiyi Tang, Qi Yu

**Affiliations:** ^1^Department of Obstetrics and Gynecology, Peking Union Medical College Hospital, Peking Union Medical College and Chinese Academy of Medical Sciences, Beijing, China; ^2^Department of Medical Research Center, Peking Union Medical College Hospital, Peking Union Medical College and Chinese Academy of Medical Sciences, Beijing, China; ^3^Laboratory of Clinical Genetics Medical Science Research Center, Peking Union Medical College Hospital, Peking Union Medical College and Chinese Academy of Medical Sciences, Beijing, China

**Keywords:** premature ovarian insufficiency, PEGylated growth hormone, reparative effect, oocyte, mitochondrion

## Abstract

Premature ovarian insufficiency (POI) is a heterogeneous disorder and lacks effective interventions in clinical applications. This research aimed to elucidate the potential effects of recombinant human PEGylated growth hormone (rhGH) on follicular development and mitochondrial function in oocytes as well as ovarian parameters in POI rats induced by the chemotherapeutic agent. The impacts of rhGH on ovarian function before superovulation on follicles, estrous cycle, and sex hormones were evaluated. Oocytes were retrieved to determine oocyte quality and oxidative stress parameters. Single-cell sequencing was applied to investigate the latent regulatory network. This study provides new evidence that a high dosage of rhGH increased the number of retrieved oocytes even though it did not completely restore the disturbed estrous cycle and sex hormones. rhGH attenuated the apoptosis of granulosa cells and oxidative stress response caused by reactive oxygen species (ROS) and mitochondrial superoxide. Additionally, rhGH modulated the energy metabolism of oocytes concerning the mitochondrial membrane potential and ATP content but not mtDNA copy numbers. Based on single-cell transcriptomic analysis, we found that rhGH directly or indirectly promoted the balance of oxidative stress and cellular oxidant detoxification. Four hub genes, Pxmp4, Ehbp1, Mt-cyb, and Enpp6, were identified to be closely related to the repair process in oocytes as potential targets for POI treatment.

## Introduction

Premature ovarian insufficiency (POI) is characterized by the loss of ovarian function before 40 years of age with primary or secondary amenorrhea, increased follicle-stimulating hormone (FSH) and reduced estradiol (E_2_), and it affects about 1% of women worldwide (Liu et al., 2014; Ghahremani-Nasab et al., 2020). Chemotherapeutic agents, which are common causes of POI, exhibit strong reproductive toxicity by promoting the abnormal activation or deletion of primordial follicles and impairing the ovarian microenvironment (Chen et al., 2019). Consequently, ovarian function and oocyte quality are severely damaged owing to cellular metabolic disorders, DNA trauma, and oxidative stress (Khedr, 2015). The currently available POI therapies, which have a limited clinical application, include assisted reproductive technology, stem cell therapy, and hormonal replacement treatment. Therefore, improved therapeutic strategies are urgently needed.

Growth hormone (GH) is a critical peptide hormone secreted by the anterior pituitary. As a member of the growth factor family, it plays a vital role in regulating growth and development, the gonadal axis, metabolism, and the psychological status (Ben-Avraham et al., 2017; Nylander et al., 2018). Recently, it has been identified to be involved in attenuating the injury caused by chemotherapy via promoting hematopoietic recovery (Zhang et al., 2008) and repairing the intestinal mucosal barrier (Ortega et al., 2001). More importantly, it has also been shown to be associated with fertility and fecundity (Satoh et al., 2002). Patients benefit from GH supplementation during controlled ovarian hyperstimulation or *in vitro* maturation, and recombinant human PEGylated growth hormone (rhGH) improves the likelihood of pregnancy by affecting the oocyte and subsequent embryo quality (Yovich and Stanger, 2010; Weall et al., 2015). A research on mice has also confirmed, in POI treatment, the crucial role of the *Notch-1* signaling pathway activated by GH at the tissue level (Liu et al., 2016). In addition, GH affects the maturation of germinal vesicle oocytes via promoting meiotic progression, balancing redox homeostasis of the microenvironment, and enhancing oocyte developmental competence (Li et al., 2019). In recent years, growing studies have observed some potential benefits under GH intervention for women with poor ovarian response (Dakhly et al., 2016; Chu et al., 2018; Cai et al., 2019), and it has already been written into the Chinese expert consensus as a recommended adjuvant therapy drug in 2015. Three studies of meta-analysis have successively evaluated the value of the usage of GH. One of the studies in 2010 in the Cochrane database concluded that GH addition could improve the clinical pregnancy rate and live birth rate (Duffy et al., 2010). Another study in 2015 revealed an increased the serum E_2_ level on the day of HCG, MII oocyte number, zygotes number with 2 pronuclei, and even obtained embryos although the implantation rate and clinical pregnancy rate did not differ after GH treatment (Yu et al., 2015). The third study in 2018 made similar conclusions besides a reduced use of gonadotropin during controlled ovarian stimulation cycles (Li et al., 2017). All these studies support the potential of GH for fertility improvement. However, most studies focused on individuals with poor ovarian response or aging, and no unified drug regimen on the timing and dosage of rhGH is available in clinical application for these patients. There is still insufficient evidence to ascertain the therapeutic effect of GH in POI, as the underlying mechanisms on oocytes remain not so clear.

Therefore, the principal objectives of this study were to investigate the therapeutic effects of the long-acting rhGH modified by drug PEGylation or polyethylene glycol recombinant human growth hormone, the structure of which is conjugated with polyethylene glycol moiety (a longer half-life, slower plasma clearance, and reduced immunogenicity than traditional GH) (Hou et al., 2016; Saenger and Mejia-Corletto, 2016) on ovarian function, especially oocyte quality, in POI rats induced by chemotherapeutic agent, and to explore the possible mechanisms associated with the improved outcomes.

## Materials and Methods

### Animals and Study Design

Five weeks old female SD rats were purchased from Beijing Vital River Laboratory Animal Technology Corporation and experiments were carried out following the National Institutes of Health’s Guide for the Care and Use of Laboratory Animals. The present study was performed with ethical approval from the Committee of Animal Experimentation of Peking Union Medical College Hospital, Chinese Academy of Medical Sciences & Peking Union Medical College. Rats were maintained for one week to aid acclimatization in specific pathogen-free conditions and were divided into five groups: (I) Wt (normal control rats); (II) Mo (rats intraperitoneally injected with 50 mg/kg cyclophosphamide (CTX) for the first time, followed by a 14-day consecutive injection of 8 mg/kg CTX to establish a POI animal model); (III) Tl (POI model rats injected with low dosage, 0.4 mg/kg rhGH); (IV) Tm (POI model rats injected with medium dosage, 0.8 mg/kg rhGH); and (V) Th (POI model rats injected with high dosage, 1.6 mg/kg rhGH). The former two groups were subcutaneously injected with PBS as a normal control or disease control. The latter three groups were subcutaneously injected with rhGH of different dosage, a long-acting agent, once per week for 28 days. Rats were housed in a temperature-controlled environment under a standard dark-light cycle and had free access to food and water. Among the experimental subjects, 12 rats from each group (60 in total) were finally used to collect tissue samples including ovaries and blood and 8 ones were arranged for oocyte induction for assessing the quality and mitochondria-relevant experiments.

### Vaginal Cytology

Vaginal smears were obtained daily by flushing with 20 μl PBS and staining with Wright & Giemsa (Solarbio Life Science, Beijing, China), mixed with PBS solution for 3–5 min and rinsed subsequently with water. The estrous cycle was defined based on the cell types (nucleated cells, non-keratinized squamous cells, and leukocytes), observed under a microscope at ×100 magnification and was distinguished into proestrus (P), estrus (E), and meta-estrus (M) or diestrus (D) (Feng et al., 2019).

### Morphological Observation and Follicle Counting

Ovaries harvested from each group were fixed with 4% paraformaldehyde overnight and then sectioned into 5 μm thick slices and mounted on glass slides for routine H&E staining for pathological analysis. To classify and enumerate follicles, 10 interval slices from one ovary were selected for each sample and only follicles containing an oocyte were counted in every slide. Follicles were classified as primary, secondary, and antral or preovulatory follicles according to the presence and distribution of granulosa cells, follicular antrum, or cumulus cells as previously described (Myers et al., 2004). Primary follicles were determined when oocytes covered with a single layer of granulosa cells of cuboidal shape. Secondary follicles were identified when there was more than one layer of granulosa cells. For antral follicles, they were characterized by an antral space formed by follicular fluid. Preovulatory follicles typically possessed a rim of cumulus cells.

### Enzyme-Linked Immunosorbent Assay

Blood samples were extracted by heart puncture and isolated after centrifugation at 1,500 g/min for 15 min. The enzyme-linked immunosorbent assay (ELISA) panels (Cusabio, Wuhan, China) were used for E_2_ and FSH detection. E_2_ and FSH standards were diluted into 40–1,000 pg/ml and 0.17–10 mIU/ml, respectively. HRP-conjugate mixed solution was added into the samples and incubated at 37°C for 1 h. After washing thrice, the substrate was added into each well. The plate was kept away from drafts in the dark, followed by stop solution reaction. The kits used employed a competitive inhibition enzyme immunoassay technique, and the final results of optical density of each well were measured using a microplate reader set to 450 nm.

### Apoptosis Assay

Ovarian slices were incubated with TUNEL FITC Apoptosis Detection Kit (Vazyme, Nanjing, China). Tissue permeability was enhanced by treatment with 20 μg/ml proteinase K for 10 min at room temperature. The slices were rinsed with PBS thrice for 5 min. For labeling and detection, slices were firstly covered with equilibration buffer for 20 min. Samples were then incubated with terminal deoxynucleotidyl transferase and FITC-12-dUTP Labeling Mix buffer in the dark. After washing with PBS, slices were further rinsed with clearing solution with 0.1% Triton X-100 and 5 mg/ml BSA. Tissue sections were observed for apoptotic cells with fragmented nuclei using a fluorescence microscope.

### Quantitative Reverse Transcription Polymerase Chain Reaction Analysis

mRNA levels of respiratory-chain-related genes were determined using quantitative reverse transcription polymerase chain reaction (qRT-PCR), and total RNA was extracted from ovarian tissues with TRIzol Reagent (Takara, Japan). Reverse transcription was performed to generate cDNA strand preconditioned with gDNA Eraser (Takara, Japan). qRT-PCR was conducted using SYBR^®^ Premix Ex Taq^TM^ (Takara, Japan). PCR primer sequences were designed using the primer premier v6.0 software and blasted using Primer-Blast as listed in Table 1. Cycling conditions for the PCR machine were set as follows: 95°C for 3 s and 60°C for 30 s for 40 cycles in a 20 μl reaction volume. Gene amplification levels were quantified by the delta-delta CT method and standardized to that of the reference gene.

**TABLE 1:** Primers information.

Gene		
*Ndufs3*	Forward	GTATGAGAGGGAGGTCTGGGA
	Reverse	AGGGGCTGTTCAGGTCAAAC
*Sdha*	Forward	AAGCTCCTGCCTCCGTGGTT
	Reverse	AGCGACACAGCAACACCGATG
*Uqcrc2*	Forward	CAACAACACCACCAGCCTCCT
	Reverse	ATAACATCTCCAGCCGCAGCAG
*Atp5f1a*	Forward	TAATGGCAAGCACGCTCTGA
	Reverse	AGCAGGCGAGAGTGTAGGTA
β*-Actin*	Forward	CTGTCCACCTTCCAGCAGATGT
	Reverse	GCTCAGTAACAGTCCGCCTAGA

### Ovulation Induction and Oocyte Incubation

Ovulation was induced as previously described, with modifications (Cornejo-Cortes et al., 2006). Briefly, after four-week GH treatment, rats were super-ovulated with 50 IU pregnant mare serum gonadotropin (ProSpec, Israel). After 48 h, 50 IU human chorionic gonadotropin (ProSpec, Israel) was administered intraperitoneally to trigger oocytes maturation. Oviducts were harvested 24 h after hCG administration. Oocytes were then released into pre-heated G-MOPS^plus^ medium (Vitrolife, Sweden) by tearing oviducts with a needle. Cumulus-free oocytes were harvested from the oocyte-corona-cumulus complex after removing granulosa cells with the addition of 0.3 mg/ml hyaluronidase (Sigma-Aldrich). They were washed thrice with G-MOPS^plus^ and finally incubated at 37°C in G-IVF^plus^ medium (Vitrolife, Sweden).

### Evaluation of Oocyte ATP Content

Each oocyte was transferred into a sterile tube with 100 μl lysis solution, as an independent sample, to release ATP from the oocytes. The average ATP content in each sample and the luminescence intensity were linearly dependent. According to the kit’s guide (Beyotime, Beijing, China), a standard curve including eight ATP concentrations, ranging from 1 to ∼5000 nM, was prepared for the analysis. Samples were immediately disposed to avoid enzymatic ATP hydrolysis, which were transferred onto a 96-well plate with 100 μl detection working fluid, and the ATP concentration was finally evaluated using a luminometer.

### Mitochondrial Membrane Potential Assay With JC-1

The mitochondrial membrane potential (ΔΨm) was quantified as previously reported (Wu et al., 2009). The mitochondrial probe, JC-1 is a dual-emission and potential-sensitivity indicator for mitochondrial membrane potential that accumulates preferentially within the mitochondrial matrix to form J-aggregates (red fluorescence) when the potential is high, and conversely, disaggregates into monomers (green fluorescence) at a low potential. The oocytes retrieved were incubated in medium containing JC-1 at 37°C for 20 min (Beyotime, Beijing, China). After incubation, oocytes were rinsed with precooled JC-1 staining buffer solution twice and then transferred into confocal dishes with fresh medium. Finally, the distribution of JC-1 was determined using a confocal laser microscope. Captured images were processed using Image J software.

### Quantification of Mitochondrial DNA Copy Number

Oocytes were transferred individually to a 200 μl tube containing 10 μl lysis buffers (50 mM Tris-HCl, 0.1 mM EDTA, 0.5% Tween-20 and 200 mg/ml proteinase K) and incubated at 55°C for 20 min and then at 95°C for 10 min sequentially to release the DNA. qPCR primers for rat mtDNA sequences (*mtND2*) (forward primer: CCTCATAGGGCCTGTAATCACT; reverse primer: GCTGCTTCAGTTGATCGTGG) were designed. Absolute quantification of the mtDNA copy number was performed, and template DNA was extracted from oocytes for the construction of the pESI-T vector, linked with *mtND2* gene and validated with Sanger sequencing (Takeo et al., 2014, 2015; Hou et al., 2018). qRT-PCR was then performed to quantify the copy number of *mtND2*. PCR conditions were as follows: 95°C for 5 min and 40 cycles at 95°C for 10 s and 60°C for 30 s. A standard curve was plotted with eight 10-fold serial dilutions of the external standard for the determination of the mtDNA copy number.

### Reactive Oxygen Species and MitoSOX^TM^ Detection

Mitochondrial superoxide is the predominant reactive oxygen species (ROS) in mitochondria, generated as a byproduct of oxidative phosphorylation. It was assessed using a superoxide indicator, MitoSOX^TM^ following the manufacturer’s instructions. Briefly, oocytes were incubated in 5 μM MitoSOX^TM^ reagent working solution (Invitrogen) dissolved in the medium. Loaded cells were incubated for 10 min at 37°C in the dark and observed under a confocal microscope. Intracellular ROS was labeled by a fluorescence probe, DCFH-DA (Beyotime, Beijing, China). As recommended, live oocytes were cultured with 10 μM diluted DCFH-DA for 20 min at 37°C. Cells were then rinsed with medium thrice and evaluated using the confocal microscope.

### Single-Cell RNA Sequencing and Bioinformatic Analysis

Every individual oocyte was placed into sterile 200 μl tubes for full-length mRNA amplification and DNA library construction for Illumina sequencing. Based on the results from the experiments above, oocytes were retrived from the group with the optimal dosage of rhGH (1.6 mg/kg) for further single-cell RNA sequencing analysis. Thus, a total of 89 cells from the Wt, Mo, and Th groups were collected and lysed with RNase inhibitor to release RNA, which were subsequently amplified to synthesize first-strand cDNA (PCR conditions were as follows: 42°C for 90 min and 70°C for 15 min) and the full-length cDNA depending on the template-switching activity of the reverse transcriptase (PCR conditions were as follows: 98°C for 1 min; 18 cycles at 98°C for 10 s, 65°C for 15 s, and 72°C for 6 min; 72°C for 5 min). Amplification products were purified using magnetic beads and evaluated with an Agilent 2100 bioanalyzer to determine the cDNA size distribution. To construct sequencing libraries for the Illumina platform, the transposase method was applied for “tagmentation” of the input (1 ng cDNA of initial mass), followed by strand displacement with adaptors and a limited-cycle PCR. Finally, the fragment library was purified using beads, and DNA molecules with lengths ranging from 300 to 700 bp were selected and quantified using Qubit according to the manufacturer’s instructions.

To determine the expression levels of genes among the groups, routine bioinformatic analysis was carried out, including quality control by FastQC and filtering of raw data to obtain high-quality clean reads were quantified based on the alignment of sequence data against the rat reference genome (Rattus_norvegicus.Rnor_6.0.dna) using Hisat2. The aligned data were further calculated to evaluate the distribution of the reads using the CollectRnaSeqMetrics module of Picard. The expected number of Fragments Per Kilobase of transcript sequence per Millions base pairs sequenced (FPKM) method was applied to calculate the expression level of genes in each oocyte. Differentially expressed genes (DEGs) were then selected by limma package when meeting the criteria *P* < 0.05 and fold-change >1.5.

### Statistical Analysis

Data were represented as mean ± SD. One-way analysis of variance was applied to evaluate significance using IBM SPSS Statistics software, version 22.0. Shapiro–Wilk test, combined with normality plots, was applied to determine the normal distribution. Results were recognized as statistically significant when *P* < 0.05.

## Results

### Effects of rhGH Treatment on the Ovarian Function Affected by CTX

**FIGURE 1:**
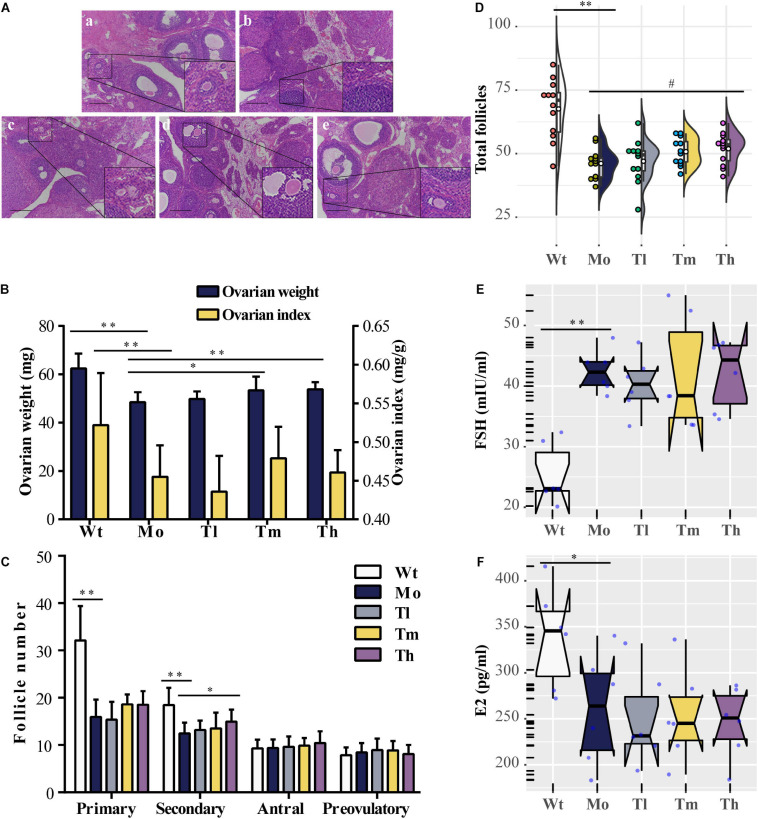
Effects of rhGH on ovarian function during POI treatment. **(A)** Hematoxylin-eosin staining for the ovarian tissues (**a, b, c, d,** and **e** for the Wt, Mo, Tl, Tm, and Th groups, respectively. *n* = 12, scale bar = 200 μm, ×100 magnification). **(B)** Ovarian weight (midnight-blue part) and ovarian index (yellow part) for each group (*n* = 12, ^∗^*P* < 0.05, ^∗∗^*P* < 0.01). **(C)** Different follicles including primary, secondary, antral, and preovulatory follicles counted after treatment (*n* = 12, ^∗^*P* < 0.05, ^∗∗^*P* < 0.01). **(D)** Half violin plot showed the total follicles from ovary enumeration (*n* = 12, ^#^*P* = 0.08, ^∗∗^*P* < 0.01). Each dot represented a sample. **(E,F)** Notched boxplots recorded the concentrations of FSH and E_2_ in rat serum of the five groups, detected using ELISA kits. The nested scatter plot as well as the rug plot in the y-axis represented each sample (*n* = 6).

Rats were sacrificed after rhGH administration for further analysis. To evaluate the reparative actions of rhGH on ovarian function, we analyzed the pathologic changes, follicles development, estrous cycle, and serum hormones of the rats. The data demonstrated, as shown in Figure 1, that CTX could cause follicle depletion and organic lesions to a certain degree with reduced total follicles, especially primary or secondary follicles (Figures 1A,C,D), and ovarian index (0.45 ± 0.04 versus 0.52 ± 0.07 for the Mo and Wt groups, respectively) or ovarian weight (Figure 1B). After therapy, the ovarian weight of the rats in the Th and Tm groups increased; however, the ovarian index remained unchanged owing to the body of rats growing at the same pace. Additionally, the results also implied that high dosage of rhGH could promote the development of the surviving primary follicles to secondary follicles (Figure 1C). However, the number of total follicles showed no statistical difference among the groups (Figure 1D). We observed a hormonal disruption in the POI model, with increased FSH (42.50 ± 3.50 versus 25.4 ± 5.02 for the Mo and Wt groups, respectively. *P* < 0.01) and decreased E_2_ (260.40 ± 60.14 versus 338.67 ± 54.77 for the Mo and Wt groups, respectively. *P* < 0.05) levels; however, rhGH therapy did not reverse the condition (Figures 1E,F). To determine the estrous cycle, vaginal smears were performed daily until the animals were sacrificed. Typical images (proestrus, estrus, and meta-estrus, or diestrus) and the records of daily estrous cycles for each group were obtained (Supplementary Figures 1A,B). As expected, the estrous cycle of rats in the Mo group revealed an anomaly, which was that it was liable to be arrested in the diestrus stage when compared with that of the normal group (Supplementary Figure 1C).

### The Modulation of the Ovarian Microenvironment by rhGH

**FIGURE 2:**
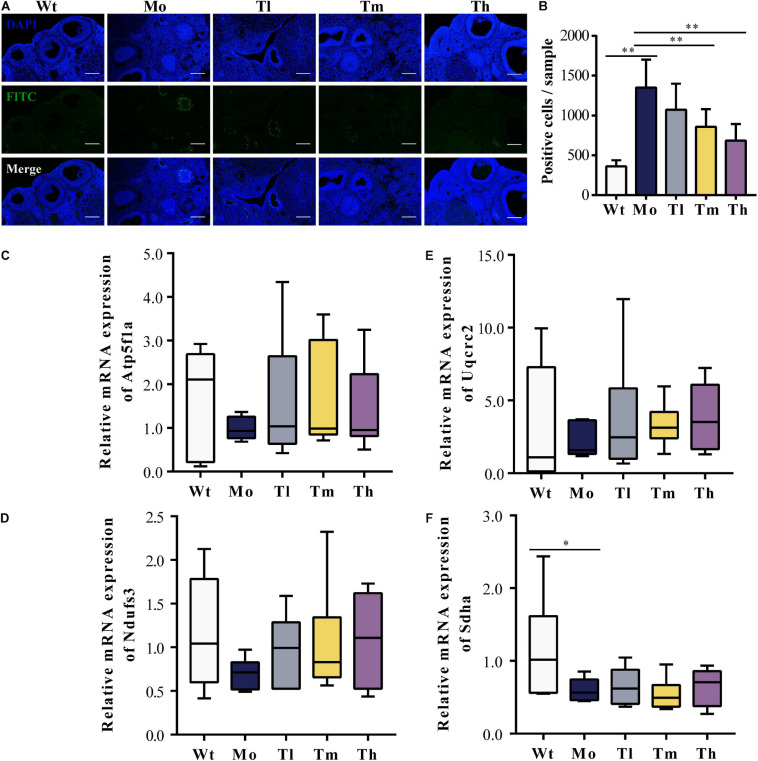
Improvement of the ovarian microenvironment after rhGH administration. **(A,B)** TUNEL assay for evaluation of the apoptosis of ovarian cells. The nuclei were labeled with DAPI staining (blue) and fragmented DNA were stained with FITC-12-dUTP (green), quantified by counting the number of FITC-labeled positive cells (*n* = 6, ^∗∗^*P* < 0.01, scale bar = 200 μm, ×100 magnification). **(C–F)** Expression levels of respiratory-chain-related genes, *Atp5f1a, Ndufs3, Sdha*, and *Uqcrc2*, respectively (*n* = 6, ^∗^*P* < 0.05).

To evaluate the ovarian microenvironment, the TUNEL assay was conducted to determine the cellular apoptosis in ovarian tissue, and the expression levels of respiratory-chain-related genes including *Atp5f1a, Ndufs3*, *Sdha*, and *Uqcrc2* were detected by qRT-PCR. Our results suggested that CTX injection clearly promoted the apoptosis of ovarian cells, mainly the granulosa cells, which played an indispensable role in follicular development by communicating directly or indirectly with oocytes and secreting various cytokines to support homeostasis in the ovarian environment. In contrast, rhGH eliminated these negative effects by protecting granulosa cells from chemotherapeutic toxicity. As shown, medium and high dosage of rhGH could apparently reduce the number of labeled positive cells or apoptotic cells (*P* < 0.01) (Figures 2A,B). *Sdha* was significantly down-regulated in the POI animal model groups compared to that of the Wt group (0.60 ± 0.16 versus 1.16 ± 0.71 for the Mo and Wt groups, *P* < 0.05); however, rhGH administration did not affect the expression level of this gene (Figure 2F). *Atp5f1a, Ndufs3*, and *Uqcrc2* showed similar variations, but without statistical significance (0.99 ± 0.27 versus 1.69 ± 1.22, 0.70 ± 0.18 versus 1.16 ± 0.65, and 2.17 ± 1.17 versus 3.13 ± 4.10 for the Mo and Wt groups, respectively) (Figures 2C–E). These results hinted that rhGH might act on attenuating the toxicity of CTX by mainly reducing the apoptosis of ovarian tissue, potentially improving oxidative metabolism, and thus modulating the ovarian microenvironment.

### Improved Quantity and Quality of the Retrieved Oocytes by rhGH

**FIGURE 3:**
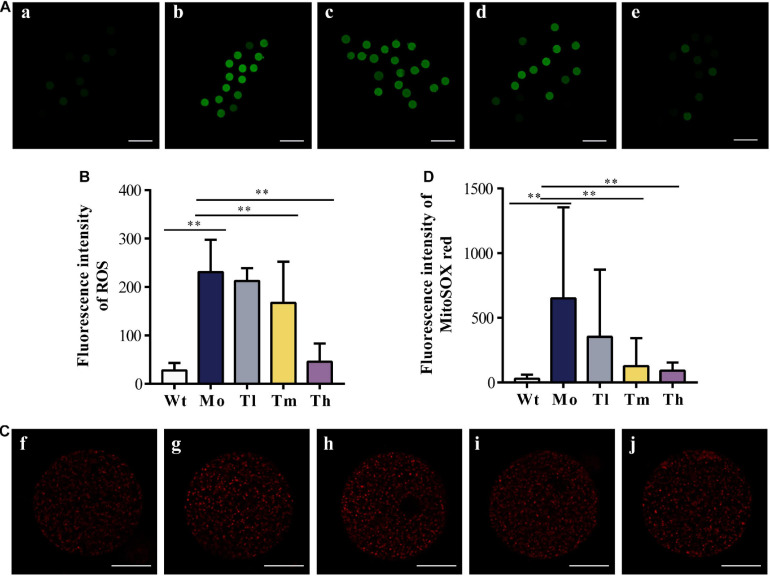
Oxidative stress response to CTX and rhGH treatment. **(A,B)** Representative images of the contents of ROS (reflected by the intensity of green fluorescence. **a, b, c, d,** and **e** for the Wt, Mo, Tl, Tm, and Th groups, respectively. *n* ≥ 9, ^∗∗^*P* < 0.01, scale bar = 200 μm, ×40 magnification) from each group and quantitative analysis shown as the histogram. **(C,D)** Typical images of the contents of mitochondrial superoxide (reflected by the intensity of red fluorescence. **f, g, h, i,** and **j** for the Wt, Mo, Tl, Tm, and Th groups, respectively. *n* ≥ 8, ^∗∗^*P* < 0.01, scale bar = 25 μm, ×400 magnification).

After ovulation induction, oocytes from each group were counted. As observed, the number of retrieved oocytes decreased dramatically (29.9 ± 6.4 for the Wt group and 16.6 ± 3.1 for the Mo group, *P* < 0.01) and a high dosage of rhGH could increase the number of oocytes obtained (20.9 ± 3.3, *P* < 0.05, compared with the Mo group) as shown in Table 2. To determine whether rhGH could improve oocyte quality, ROS and mitochondrial superoxide levels were measured in the retrieved oocytes from all groups. Results revealed that medium and high dosage of rhGH could both remarkably reduce these oxidative products when comparing with the model group (for ROS, 230.69 ± 66.88, 167.08 ± 85.06 and 45.45 ± 37.59; for mitochondrial superoxide, 650.61 ± 704.12, 126.72 ± 216.97 and 91.35 ± 63.66 for Mo, Tm and Th groups, respectively. *P* < 0.01). Conversely, CTX treatment would observably accumulate plenty of harmful agents, proved by ROS (Figures 3A,B) and MitoSOX^TM^ indicators (Figures 3C,D).

**TABLE 2:** Retrieved oocytes after ovulation induction.

Group (*n* = 8)	Wt	Mo	Tl	Tm	Th
Number	29.9 ± 6.4	16.6 ± 3.1	17.5 ± 4.2	19.5 ± 2.9	20.9 ± 3.3
*P*-value	<0.01^a^	–	0.68^b^	0.18^b^	0.05^b^

*^*a*^Comparison between the Wt and Mo groups. ^*b*^Comparisons between the Mo and three rhGH-treated groups.*

**FIGURE 4:**
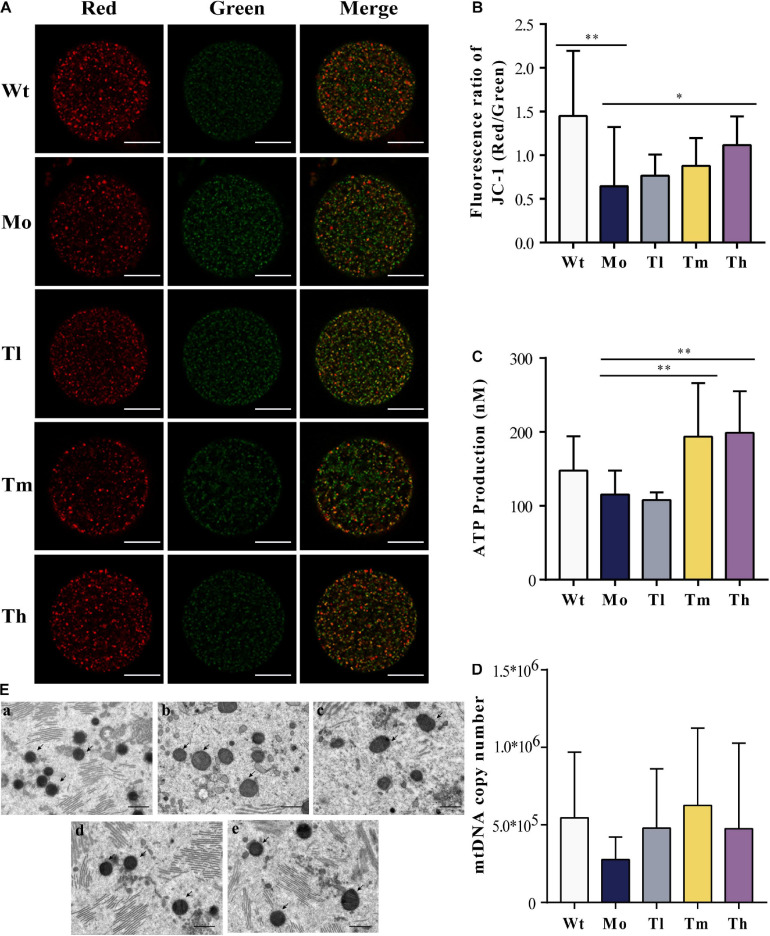
Mitochondrial quality and energy metabolism. **(A,B)** Mitochondrial membrane potential quantification, where J-aggregates or monomers were indicated by red or green fluorescence (*n* ≥ 10, ^∗^*P* < 0.05, ^∗∗^*P* < 0.01, scale bar = 25 μm, ×40 magnification). **(C)** ATP content of each group (*n* = 10, ^∗∗^*P* < 0.01). **(D)** mtDNA copy number in each oocyte (*n* = 10). **(E)** Mitochondrial status was evaluated under the electron microscope (**a, b, c, d,** and **e** for the Wt, Mo, Tl, Tm, and Th groups, respectively. Black arrows pointed to mitochondria. *n* = 8, scale bar = 10 μm, ×5,000 magnification).

Furthermore, we detected the mitochondrial membrane potentials by quantifying JC-1 either in the form of J-aggregates or monomers using the ratio of the red/green fluorescence intensity of every single oocyte from each group (Figure 4A). Our results showed that the intensity in the Wt group (1.45 ± 0.75) was more than twice that of the Mo group (0.66 ± 0.68) (Figure 4B). After treatment with high dosage of rhGH, the mitochondrial membrane potentials of oocytes in the group Th differed significantly (1.12 ± 0.33, *P* < 0.05). Regarding the ATP content, we found that medium and high dosage of rhGH could significantly promote ATP content (either increased energy production or decreased ATP consumption) (*P* < 0.01) when compared with that of the Mo group, even though no statistically significant difference was observed between the Wt and Mo groups (Figure 4C). We estimated the mitochondrial DNA copy number through the absolute quantification of the expression levels of *ND2*. The results varied greatly among oocytes, and there were no apparent differences among all groups. However, we noticed that the mitochondrial DNA copy number in the Mo group was relatively lower than that in the Wt group and that rhGH treatment did not significantly reverse the situation even though there exerted a possible improvement but without statistical difference (Figure 4D). In addition, we qualitatively evaluated the morphological changes of mitochondria, and the results showed that the organelles in the Mo group were swollen and enlarged with vague cristae, and with lower electron density in the matrix when compared to those of the Wt group. In the Tl group, the mitochondria did not differ much from the model group considering that the mitochondrial swelling was not well improved. In contrast, mitochondrial morphology changed significantly in the Tm and Th groups when compared with the Mo group, with a more uniform matrix and improved electron density (Figure 4E).

### Single-Cell Transcriptomics Analysis for the Differential Expression of mRNA

**FIGURE 5:**
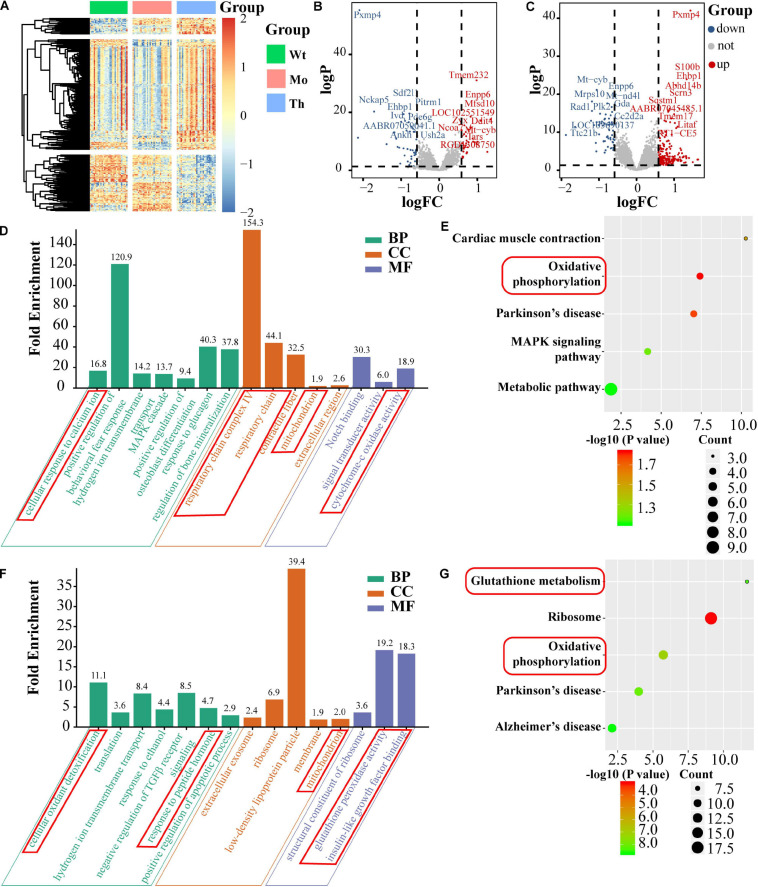
Single-cell mRNA sequencing and bioinformatics analysis. **(A)** Heatmap of DEGs. **(B,C)** Volcano plots of DEGs (left panel for comparison between the Mo and Wt groups; right panel for comparison between the Th and Mo groups); the top 10 up-regulated and down-regulated DEGs were labeled with their names. BP, CC, and MF stand for biological process, cellular component and molecular function, respectively. **(D,E)** GO enrichment [(left panel), including biological process, molecular function and cellular component] and KEGG pathway (right panel) of DEGs and the core parts were marked with red frame, comparing the Mo group with Wt group. **(F,G)** Comparison between the Th and Mo groups as described above.

To confirm and explore the potential molecular mechanisms of the cytotoxic effects of CTX and the protective effects of rhGH on oocytes, we further identified the DEGs, based on single-cell full-length mRNA sequencing technology for each oocyte from the Wt, Mo, and Th groups, considering that rhGH effects mainly occurred at high dosage. The heatmap in Figure 5A showed the relative expression of genes among groups. Among these genes, 44 were down-regulated and 34 up-regulated in the POI model group, compared with the Wt group, and the top 10 DEGs were labeled (Figure 5B). Besides, 217 genes were up-regulated, and 58 genes were down-regulated after rhGH treatment, when compared with the Mo group (Figure 5C). Several genes among the top DEGs varied with the opposite trends after CTX treatment and after rhGH intervention (the Mo group versus the Wt group; the rhGH treatment group versus the Mo group) including *Pxmp4*, *Ehbp1*, *Mt-cyb*, and *Enpp6.* Following this analysis, we identified all the DEGs based on the inclusion criterion. The DEGs were screened for further functional enrichment, and DAVID online database was utilized to determine gene functions and obtain a visualization of the results. As for GO analysis and KEGG pathways for CTX treatment compared with the normal group, we observed some items that were closely associated with energy metabolism such as cellular response to calcium ion in the biological process category; respiratory chain complex IV, respiratory chain or mitochondrion in the cellular component category; and cytochrome-c oxidase activity in the molecular function category (Figure 5D). Additionally, oxidative phosphorylation was marked by a red frame as a core modulatory signal (displayed in Figure 5E). After rhGH intervention, similar changes caused by rhGH or its downstream metabolites were detected, with cellular oxidant detoxification and response to peptide hormone in the biological process category. Additionally, the mitochondrion was the main cellular component for these DEGs. In the molecular function category, glutathione peroxidase activity, and insulin-like growth factor binding played a crucial part (Figure 5F). As expected, glutathione metabolism and oxidative phosphorylation pathways were enriched after rhGH therapy, as shown in Figure 5G. Furthermore, protein–protein interaction (PPI) analysis using the online analytical database, STRING was conducted to clarify the interactive relation among DEGs, the results of which were further visualized using Gephi software (Supplementary Figure 2A). All the DEGs from the above-mentioned modules were selected and labeled with the same color and size. The connecting lines represented the interactive relationship. The results showed, similar to those described above, that oocytes were severely affected by the CTX agent as evidenced by aberrant mitochondrial function, and that rhGH, directly or through its metabolites, improved the detoxification competence of oocytes with improved energy supply, thus restoring oocytes status.

### HubGenes Identification and Modulation Network Establishment

To identify the hub genes of the gene regulatory network structure, incorporated with prior knowledge about the DEGs, lasso-penalized regression in combination with generalized linear regression analysis was applied for integration analysis. In DEGs of the Mo group versus the normal control group, four genes were identified based on lasso-penalized analysis (Supplementary Figures 3A,B), namely *Tmem232*, *RGD1308750*, *Sdf2l1*, and *Pxmp4*, the expression levels of which were presented in a heatmap (Supplementary Figure 3C). At the same time, to uncover the connections among the four hub genes, we calculated the correlation coefficient among all the genes, and the results showed that genes shared a significant correlation (*P* < 0.05) as shown in Supplementary Figure 3D. After filtration, we plotted a nomogram based on the generalized linear regression analysis to predict the chance of POI. The higher the total number of points, the higher the chance (Supplementary Figure 3E). Consistent with the nomogram, a forest plot illustrated the odds ratio of the whole set of genes for POI chance. Sdf2l1 and *Pxmp4* were shown to act as protective factors, and the remaining genes as hazardous factors that might increase the chance (Supplementary Figure 3F).

**FIGURE 6:**
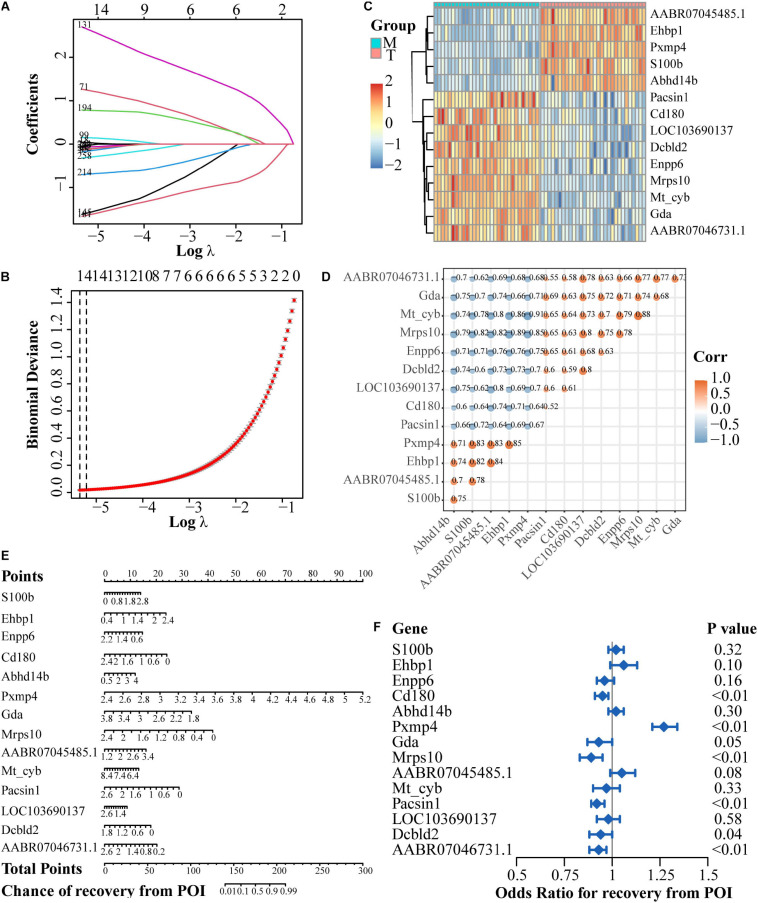
Regulatory network establishment based on DEGs after rhGH treatment, compared with the model group. **(A)** Lasso coefficient profiles of the rhGH-treatment-related mRNAs. Each curve corresponded to a gene. **(B)** Partial likelihood deviance was calculated by the cross-validation for the best lambda to determine the minimum mean cross-validated error. **(C)** Expression heatmap of the 14 hub genes. **(D)** Expression correlation among the 14 genes. Color and size of dots indicated the degree of relevance among each gene (in other words, the larger the size, the higher the correlation), which were retained only when the *P*-value of correlation coefficient was less than 0.05. **(E)** Nomogram of the hub genes. The total points were applied for evaluating the possibility of the improvement of oocyte quality. **(F)** Forest plot for the odds ratio for recovery from POI of the 14 hub genes, selected by regression analysis.

In the case of DEGs for the treatment group versus the model group, 14 genes were finally screened (Figures 6A,B). Among them, five genes were up-regulated after rhGH treatment (*AABR07045485.1*, *Ehbp1*, *Pxmp4*, *S100b*, and *Abhd14b*) and nine genes were down-regulated (*Pacsin1*, *Cd180*, *LOC103690137*, *Dcbld2*, *Enpp6*, *Mrps10*, *Mt-cyb*, *Gda*, and *AABR07046731.1*) as presented in the heatmap with expressional correlation (Figures 6C,D). Next, a nomogram and forest plot were also obtained based on the regression analysis. The total points to predict the chance of recovery from POI were calculated according to the cumulative points corresponding to gene expression level and the results indicated that some hub genes such as *Pxmp4*, *Gda*, *Mrps10*, *Ehbp1*, *Cd180*, and *Pacsin1*, were related to the progression and recovery of POI, acting as latent prognostic factors (Figures 6E,F).

## Discussion

Chemotherapeutic agents pose a threat to ovarian function, depending on the drug type and dosage (Chen et al., 2010; Roness et al., 2014). To date, the agents or adjuvants used to assist POI patients in fertility recovery or preservation are limited in clinical application. The decline in the oocyte quality caused by POI is a crucial factor affecting female fertility, which is often accompanied by abnormal quantity and dysfunction of oocyte mitochondria (Zhu et al., 2020). Growing studies have supported the perspective that mitochondria are vital indicators of oocyte quality (Schatten et al., 2014; Qi et al., 2019). Not only can mitochondrion, acting as an intracellular energy factory, provide ATP for oocyte maturation and fertilization, but it will also interact with the nucleus in multiple crucial biological processes, which are essential for oocyte competence and early embryo development or implantation by promoting the formation and the maintenance of spindles fertilization (Benkhalifa et al., 2014; Matilainen et al., 2017). On the contrary, disrupted mitochondrial function has been verified to cause oocyte aging (Hou et al., 2018). Recent studies have shown that GH can reverse senescence and improve the reproductive outcomes (Hou et al., 2018; Fahy et al., 2019). However, whether this will be a promising agent in protecting ovarian function and oocyte development competence from POI damage remains unclear.

As previously reported, CTX was shown to be detrimental to follicle development by inducing apoptosis of granulosa cells, disturbing the indispensable environment for oocyte maturation, and finally causing prolonged and severe damage to ovaries (Song et al., 2016; Pascuali et al., 2018; Peng et al., 2019). Consistent with these studies, we found that CTX seriously affected ovarian structure and function by promoting excessive activation and subsequent deletion of oocytes with decreased primary or secondary follicles and disrupted estrous cycles (Fu et al., 2017; Hou et al., 2018). Also, we investigated the effects of CTX on endocrinology function by examining serum FSH and E_2_. E_2_ originated from granulosa cells is regulated under the control of FSH and aromatase. The decreased E_2_ level in the blood will, in a feedback loop, stimulate the pituitary gland to secrete more FSH. Currently, we explored the effect of rhGH on POI. Growing studies have revealed the potential role of rhGH treatment or pretreatment during IVF/ICSI cycles in patients with aging, poor ovarian response or even polycystic ovary syndrome (Bayoumi et al., 2015; Lan et al., 2019; Gong et al., 2020). We did not observe significant changes in these phenotypes after treatment, but rhGH indeed increased the number of retrieved secondary oocytes at the high dosage and increased ovarian weight at the medium and high dosages. This result is encouraging. Once patients with POI can benefit from rhGH intervention to gain more oocytes during IVF/ICSI cycles, it will possibly increase the chance of pregnancy or live birth, which requires further clinical verification. Apart from these direct indicators of ovarian function, we also noticed that the microenvironment associated with oocyte maturation changed considerably after rhGH administration. As reported, CTX could trigger great cytotoxicity on either mitotic non-luteinized or non-mitotic luteinized granulosa cells *in vitro* (Yuksel et al., 2015) and it would induce the activation of some proapoptotic gene, ROS generation as well as glutathione depletion (Tsai-Turton et al., 2007). Here, we also noticed that rhGH intervention could attenuate CTX-induced the apoptosis of granulosa cells, especially for medium and high dosage of rhGH. Follicles are the basic units of ovarian function, and granulosa cells are essential for oocyte development, maturation, ovulation, and atresia (Gilchrist et al., 2004) and connect with oocytes through intercellular connection, nutritional support or direct regulation of follicular microenvironment as an organic whole (Su et al., 2009). CTX also inhibited respiratory chain complex genes, especially *Sdha*, which codes for a flavoprotein subunit of the succinate dehydrogenase complex in the mitochondrial respiratory chain (Pantaleo et al., 2014). Together with that of the other three genes (*Atp5f1a, Ndufs3*, and *Uqcrc2*), *Sdha* expression showed similar trends, suggesting that an imbalance of oxidative metabolism homeostasis may be involved in the disturbance of ovarian function. In the present study, we did observe increased trends, to some extent, of the expression level of these respiratory chain complex genes in ovaries after rhGH treatment but without statistical difference. These results hinted that the improvement of oxidative metabolism of the whole ovaries in POI rats might be a potential target of rhGH intervention and it is indeed necessary to analyze in depth with more convincing evidence. Additionally, rhGH in this research was proved to be unable to directly recover the damaged ovarian function in POI rats to an ideal status but it might come into effect in an indirect way by eliminating the apoptosis of granulosa cells, indispensable for follicular development.

Our results showed that a high dosage of rhGH increased the number of retrieved oocytes, and that medium and high concentrations of rhGH even reduced the accumulation of reactive oxidative products. Mammalian oocytes are extremely sensitive to ROS, and an imbalance of intracellular oxidation and antioxidation can disrupt oocyte development and maturation or even fertilization (Duan et al., 2015). Additionally, ATP consumption is an indispensable process from growth to fertilization of oocytes. ATP synthesis is highly correlated with impaired oocyte maturation and even embryonic development after fertilization, and strongly dependent on mitochondrial membrane potential and the quality and copy number of the organelle (Wilding et al., 2001; Van Blerkom et al., 2006). Therefore, in the present study, we explored the effects of different concentrations of rhGH on the mitochondria (Wang et al., 2019). As expected, high dosage of rhGH sustained the mitochondrial membrane potential, and medium and high concentrations of rhGH promoted ATP content to meet the energy supply requirements for oocytes. Our results also indicated that rhGH improved mitochondrial function mainly by changing the mitochondrial morphology. Mitochondrial DNA copy number in oocytes has been reported to be correlated with the developmental potential of oocytes. Consistent with previous reports, the mitochondrial DNA copy number varied, but showed a possible trend to increase with higher concentrations of rhGH. These results fully support the notion that rhGH directly or indirectly modulates the oocyte quality mainly by improving the mitochondrial state and increasing the stability of the energy metabolism, thus relieving the oxidative stress in oocytes resulting from POI toxicity. Additionally, it is worth noting that high dosage of rhGH (1.6 mg/kg) will perform more effectively in most cases in POI rats when it comes to the improved outcomes related to oocytes quality in comparison with the medium or low dosage. Thus, evidence from this study is inclined to support an appropriately higher dosage in future clinical application may be more significant but waiting for sufficient clinical data.

Our single-cell full-length mRNA sequencing analysis demonstrated that CTX treatment disturbed the respiratory chain in oocytes and led to oxidative stress bursts. rhGH attenuated these deleterious effects directly or through its metabolites and promoted detoxification. Subsequently, glutathione metabolism, along with glutathione peroxidase activity was facilitated. Intracellular oxidative phosphorylation was modulated to ameliorate the situation. It is important to note that some hub genes such as *Pxmp4*, *Ehbp1*, *Mt-cyb*, and *Enpp6*, were involved in both the injury and repair processes. *Pxmp4* encodes a 24 kDa protein that belongs to the peroxisomal membrane protein Pxmp2/4 family with unclear function although some studies have established a crucial role of peroxisomes in catalyzing a series of indispensable metabolic processes such as glyoxylate detoxification, fatty acid oxidation, and ether phospholipid biosynthesis (Visser et al., 2007). The expression level of *Pxmp4* was significantly down-regulated after CTX treatment and was increased after rhGH intervention. As shown in the nomograms and forest plots, under-expression of this gene would increase the chance of POI and vice versa. However, the underlying mechanism of this gene in the reproductive system remains unclear, which has only been reported in some cancers. As for *Ehbp1*, it can encode an Eps15 homology domain-binding protein, which has much to do with endocytic trafficking to the actin cytoskeleton, endosomal tubulation and GLUT4 transport (Guilherme et al., 2004b; Wang et al., 2016). Likewise, over-expression of *EHBP1* tended to increase the chance of recovery from POI under the treatment of rhGH. *EHBP1* is known to co-localize with the actin cytoskeleton, and overexpression of *EHBP1* triggers abundant actin reorganization (Guilherme et al., 2004a). In the process of oocyte maturation, the spindle will migrate from the geometric center, as reported previously, to beneath the oocyte surface, which often depend on an actin meshwork (Sakamoto et al., 2020). The results demonstrated that rhGH might affect oocyte maturation by modulating actin reorganization to help POI rats to retrieve oocytes of improved quality. *Mt-cyb* encodes a protein which is part of the ubiquinol-cytochrome c reductase complex in the respiratory chain that participates in generating an electrochemical potential coupled to ATP synthesis. The mutation of this gene is highly related to NLRP3-inflammasome activation and can also cause severe respiratory chain enzyme deficiency (Blakely et al., 2005; Cordero et al., 2016). Defects in *Mt-cyb* are a rare etiological factor of mitochondrial dysfunction underlying different myopathies or multisystem disorder such as prominent exercise intolerance and hypertrophic cardiomyopathy (Hagen et al., 2013; Carossa et al., 2014). In this study, we found that the expression level of Mt-cyb was up-regulated after CTX treatment. As for *Enpp6, it* is involved in regulating extracellular nucleotide metabolism and producing phosphocholine from the matrix vesicle membrane (Stewart et al., 2018). The encoded preproprotein of *Enpp6* undergoes proteolysis to generate a glycosylphosphatidylinositol-anchored membrane protein, which hydrolyzes choline-containing lysophospholipids such as glycerophosphocholine (Morita et al., 2016). *Mt-cyb* and *Enpp6* altered in similar trends as the top DEGs, which might be a compensatory regulation mechanism of oocyte itself to relieve the disturbance of oxidative metabolism caused by the chemotherapeutic agent. On the whole, the four genes are closely correlated with the phenotypes of ovarian damage and fertility repair and are thus potential targets for POI treatment. The effects of these genes on the development and metabolism of oocytes need to be further studied, considering that ovaries from either animal or human beings may not response similarly to CTX and rhGH due to the metabolic difference among species. It remains to verify the consistent trends or indicative action of the four hub genes in POI animals and patients after treatment with rhGH.

## Conclusion

We conclude that long-acting rhGH is a promising intervention or adjuvant for fertility protection in POI, and mainly exerts its effects by modulating the ovarian microenvironment with the elimination of granulosa cells apoptosis and injury from oxidative stress, and directly or indirectly improving the energy metabolism or oxidative detoxification of oocytes. However, there is still an urgent need for further studies to evaluate the safety and efficacy of rhGH for future clinical application.

## Data Availability Statement

The original contributions presented in the study are publicly available. This data can be found here: https://www.ncbi.nlm.nih.gov/bioproject/?term=PRJNA692755, accession number: PRJNA692755.

## Ethics Statement

The animal study was reviewed and approved by the Committee of Animal Experimentation of Peking Union Medical College Hospital, Chinese Academy of Medical Sciences & Peking Union Medical College.

## Author Contributions

PF, QX, and ZL: Software, data curation, formal analysis and visualization. PF, ZG, and RT: writing-original draft preparation and writing-review and editing. QY: conceptualization and design, administration and funding acquisition. All authors contributed to the article and approved the submitted version.

## Conflict of Interest

The authors declare that the research was conducted in the absence of any commercial or financial relationships that could be construed as a potential conflict of interest.

## Supplementary Material

Supplementary Figure 1 Daily records of the estrous cycle. **(A)** Representative pictures of different stages of the estrous cycle: proestrus (P) **(a)**, estrus (E) **(b)**, meta-estrus (M) **(c)** and diestrus (D) **(d)**. scale bar = 200 μm, × 100 magnification. **(B)** Typical dynamic trends of the estrous cycle for each group. **(C)** Enumeration of the estrous cycle ranging from the initiation of rhGH treatment to the fourth week after therapy.

Supplementary Figure 2 Protein-protein interaction network of DEGs. **(A)** The interactive relationship was identified, and genes belonging to different functional modules were distinguished by various colors.

Supplementary Figure 3 Model establishment of DEGs after CTX treatment. **(A)** Lasso coefficient profiles of CTX-treatment-related mRNAs. **(B)** Partial likelihood deviance was calculated by the cross-validation for the best lambda to determine minimum mean cross-validated error. **(C)** Expression heatmap of the four selected hub genes. **(D)** Expression correlation among the four genes. **(E)** Nomogram of the hub genes. The total points were applied for evaluating the chance of the POI. **(F)** Forest plot odds the odds ratio of the four hub genes by regression analysis.
