# Supplementary figures and images for: Study on the Reparative Effect of PEGylated Growth Hormone on Ovarian Parameters and Mitochondrial Function of Oocytes From Rats With Premature Ovarian Insufficiency
Source: Front Cell Dev Biol. 2021 Mar 15;9:649005. doi: 10.3389/fcell.2021.649005 (PMC8005617; doi:10.3389/fcell.2021.649005)

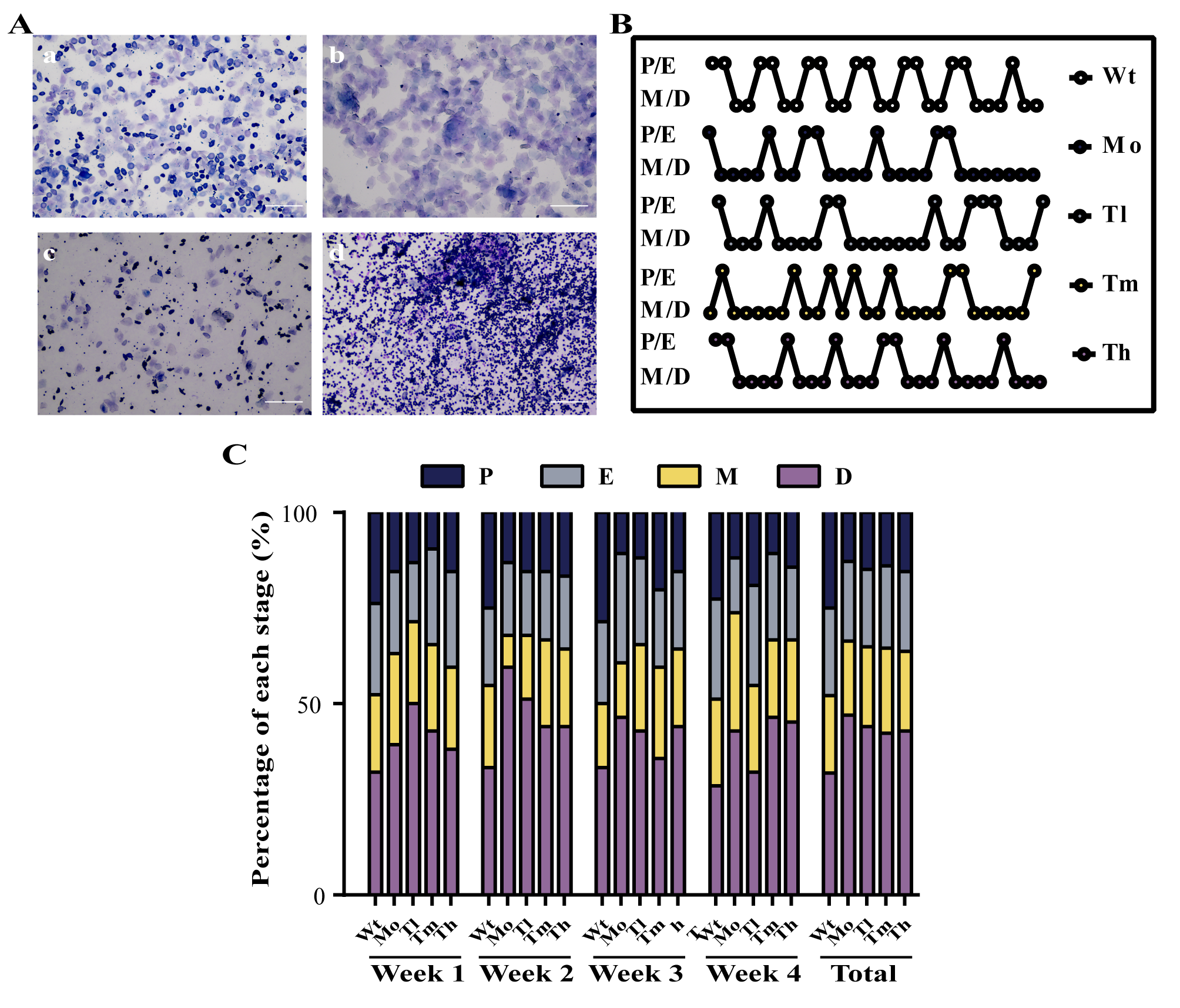

Supplement: Supplementary Figure 1 — Daily records of the estrous cycle. (A) Representative pictures of different stages of the estrous cycle: proestrus (P) (a), estrus (E) (b), meta-estrus (M) (c) and diestrus (D) (d). scale bar = 200 μm, × 100 magnification. (B) Typical dynamic trends of the estrous cycle for each group. (C) Enumeration of the estrous cycle ranging from the initiation of rhGH treatment to the fourth week after therapy. [file Image_1.TIF]

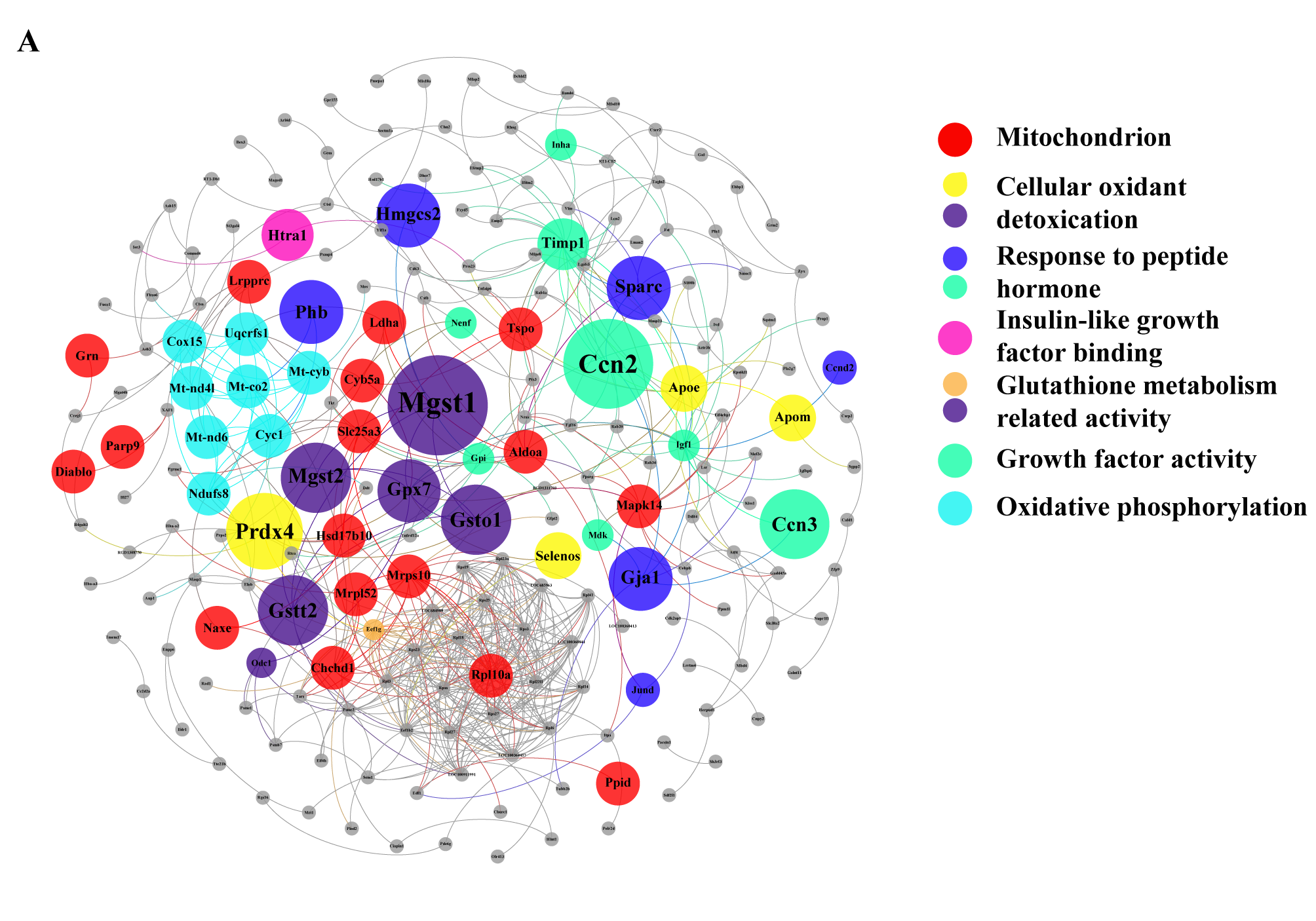

Supplement: Supplementary Figure 2 — Protein-protein interaction network of DEGs. (A) The interactive relationship was identified, and genes belonging to different functional modules were distinguished by various colors. [file Image_2.TIF]

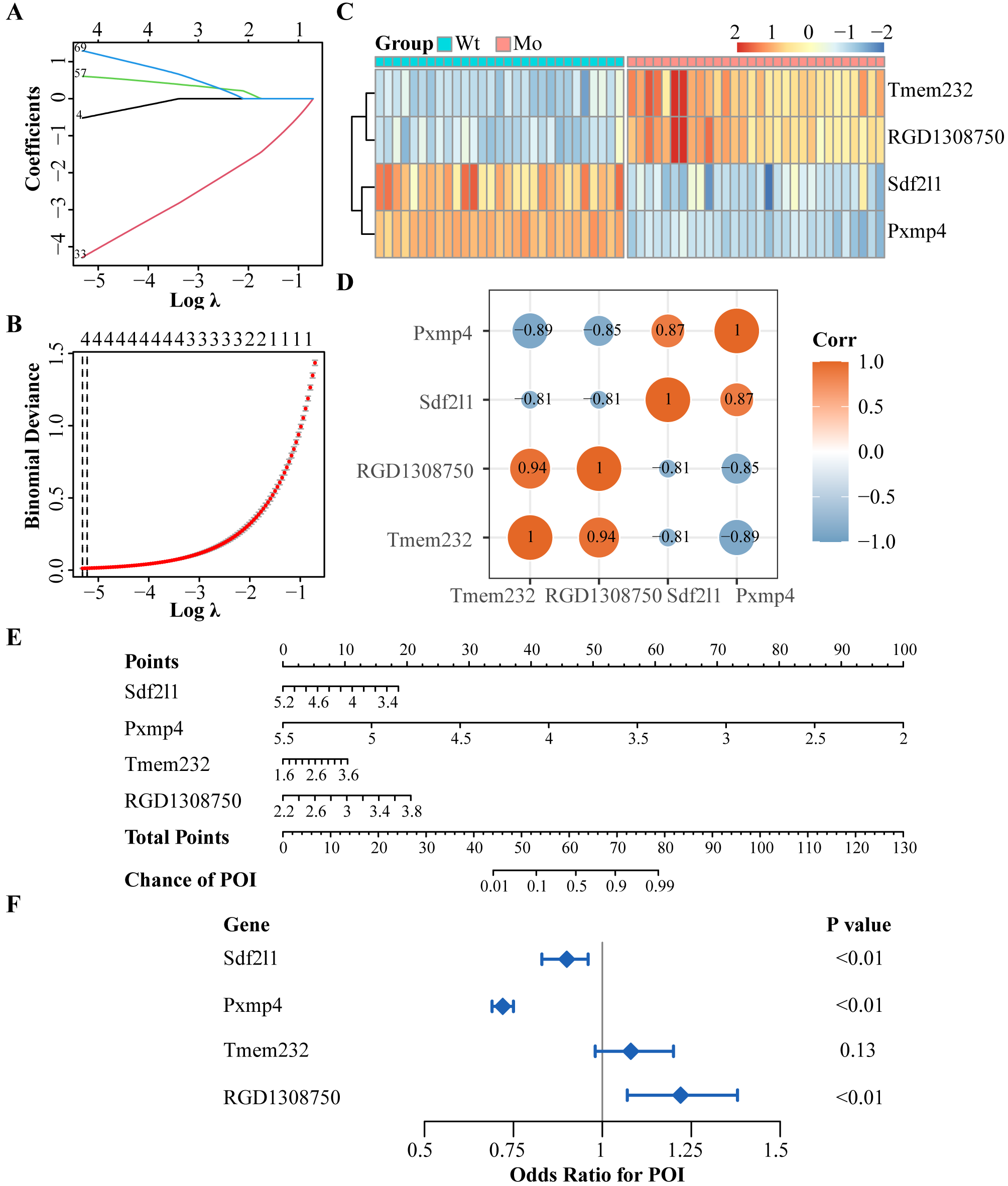

Supplement: Supplementary Figure 3 — Model establishment of DEGs after CTX treatment. (A) Lasso coefficient profiles of CTX-treatment-related mRNAs. (B) Partial likelihood deviance was calculated by the cross-validation for the best lambda to determine minimum mean cross-validated error. (C) Expression heatmap of the four selected hub genes. (D) Expression correlation among the four genes. (E) Nomogram of the hub genes. The total points were applied for evaluating the chance of the POI. (F) Forest plot odds the odds ratio of the four hub genes by regression analysis. [file Image_3.TIF]
